# Serum Osteocalcin Level is Negatively Associated with Vascular Reactivity Index by Digital Thermal Monitoring in Kidney Transplant Recipients

**DOI:** 10.3390/medicina56080400

**Published:** 2020-08-09

**Authors:** Lin Lin, Liang-Te Chiu, Ming-Che Lee, Bang-Gee Hsu

**Affiliations:** 1 Division of Gastroenterology, Hualien Tzu Chi Hospital, Buddhist Tzu Chi Medical Foundation, Hualien 97004, Taiwan; Kinase1982@yahoo.com.tw; 2 Division of Nephrology, Hualien Tzu Chi Hospital, Buddhist Tzu Chi Medical Foundation, Hualien 97004, Taiwan; carol75820@gmail.com; 3 Department of Surgery, Hualien Tzu Chi Hospital, Buddhist Tzu Chi Medical Foundation, Hualien 97004, Taiwan; 4 School of Medicine, Tzu Chi University, Hualien 97004, Taiwan

**Keywords:** osteocalcin, endothelial function, vascular reactivity index, age, kidney transplantation

## Abstract

Background and Objectives: Osteocalcin is the most abundant noncollagenous protein in bone matrix, which is considered a marker of bone formation. Previous studies indicate that circulating osteocalcin can be expressed by osteoblasts and even by osteoblast-like cells in vessel walls, and it is often associated with arterial stiffness. Our study aims to examine the potential association between osteocalcin levels and endothelial function among kidney transplant (KT) recipients. Materials and Methods: Fasting blood samples were obtained from 68 KT recipients. To measure the endothelial function and vascular reactivity index (VRI), a digital thermal monitoring test (VENDYS) was used. A commercial enzyme-linked immunosorbent assay kit was also utilized to measure serum total osteocalcin levels. In this study, a VRI of less than 1.0 indicated poor vascular reactivity; a VRI of 1.0–2.0 indicated intermediate vascular reactivity; and a VRI of 2.0 or higher indicated good vascular reactivity. Results: Our findings show that 8 KT recipients (11.8%) had poor vascular reactivity (VRI < 1.0), 26 (38.2%) had intermediate vascular reactivity (1.0 ≤ VRI < 2.0), and 34 (50%) had good vascular reactivity. Increased serum osteocalcin levels (*p* < 0.001) were found to be associated with poor vascular reactivity. Advanced age (*r* = −0.361, *p* = 0.002), serum alkaline phosphate level (*r* = −0.254, *p* = 0.037), and log-transformed osteocalcin levels (*r* = − 0.432, *p* < 0.001) were identified to be negatively correlated with VRI in KT recipients. Multivariable forward stepwise linear regression analysis revealed that the serum level of osteocalcin (β = −0.391, adjusted R^2^ change = 0.174; *p* < 0.001) and advanced age (β = −0.308, adjusted R^2^ change = 0.084; *p* = 0.005) were significantly and independently associated with VRI in KT recipients. Conclusions: Higher serum osteocalcin level was associated with lower VRI and poorer endothelial dysfunction among KT recipients.

## 1. Introduction

Cardiovascular disease is one of the major causes of morbidity and mortality in kidney transplant (KT) recipients. The annual rate of cardiovascular events is approximately 3.5% to 5%, and these events are responsible for approximately 30% of all-cause deaths [1]. This could be attributed to the presence of traditional risk factors such as diabetes, hypertension, dyslipidemia, and atherosclerosis [2]. Nontraditional factors were also found to significantly contribute to cardiovascular events. These include time on dialysis before transplantation, drugs used for immunosuppression, allograft dysfunction, anemia, and elevated homocysteine levels [2,3].

Endothelial dysfunction is a key initial event in the pathogenesis of atherosclerosis, and it contributes to the formation and progression of plaque [4]. It has often been associated with a wide range of cardiovascular diseases, including coronary artery disease and chronic heart failure, as well as chronic renal failure [4]. Various studies have demonstrated that functional changes in the arteries occur before structural changes and respond faster to therapy [5]. Endothelial dysfunction is characterized by impaired endothelium-dependent vasodilation, and it also leads to endothelial activation with proinflammatory, proliferative, and procoagulatory changes conductive to the development of atherosclerosis [6]. Vascular reactivity is a vital part of vascular function that responds to physiologic stimuli by alterations in blood flow, vessel tone, and vessel diameter [5]. Previous research has showed the association of poor vascular reactivity index (VRI) with a high Framingham Risk Score and a high level of coronary calcium score [7]. In end-stage renal disease, vascular reactivity is impaired, and such impairment is directly associated with increased rates of mortality [8].

Osteocalcin, a γ-carboxyglutamic acid containing noncollagenous protein, has been identified to be secreted by osteoblasts, and they are usually stored in bones, bound to hydroxyapatite and calcium; in its undercarboxylated form, it is released into circulation. It is now generally accepted that impaired bone metabolism may lead to the development of vascular calcification; this concept is consistent with the observation that bone loss is associated with increased vascular calcification and increased incidence of cardiovascular events [9]. Vascular smooth muscle cells play an important role in the pathogenesis of vascular calcification: they change from being contractile to being osteogenic and express osteogenic cell markers, including osteocalcin [10]. We aimed to investigate the association between osteocalcin levels and endothelial function among KT recipients.

## 2. Materials and Methods

### 2.1. Participants

A total of 68 patients, who received non-preemptive KTs at a medical center in Hualien, Taiwan, between September 2015 and February 2016, were included in this study. The study protocol was approved by the Research Ethics Committee of Hualien Tzu Chi Hospital of the Buddhist Tzu Chi Medical Foundation (IRB104-84-B) and was conducted in accordance with the tenets of the Helsinki Declaration. All patients provided their written informed consent before participating in this study. Hypertension has been defined as a systolic blood pressure of 140 mm Hg or higher or a diastolic blood pressure of 90 mm Hg or higher; patients who had received any antihypertensive medication in the previous 2 weeks were considered to have hypertension. Patients were excluded from the study if they had a dialysis access fistula or grafts in the upper extremities, acute infection, heart failure, acute transplant rejection status, and malignancy at the time of blood sampling; some opted not to join the study.

### 2.2. Anthropometric Analysis

Participants were weighed, wearing lightweight clothing without shoes, to the nearest 0.5 kg, and body height was measured to the nearest 0.5 cm. Body mass index was calculated as the weight in kilograms divided by the height in meters squared [11].

### 2.3. Biochemical Investigations

Fasting blood samples (approximately 5 mL) of all participants were immediately centrifuged at 3000 g for 10 min. An auto-analyzer (Siemens Advia 1800; Siemens Healthcare GmbH, Erlangen, Germany) was used to measure serum levels of blood urea nitrogen (BUN), creatinine, fasting glucose, total cholesterol, triglycerides, calcium, and phosphorus [11]. Commercially available enzyme-linked immunosorbent assays were also used to measure serum total osteocalcin levels (eBioscience, Inc., San Diego, CA, USA) and intact parathyroid hormone (iPTH) levels (Diagnostic Systems Laboratories, Webster, TX, USA) [12]. The estimated glomerular filtration rate (eGFR) was calculated using the Chronic Kidney Disease Epidemiology Collaboration (CKD-EPI) equation.

### 2.4. Endothelial Function Measurements

After patients fasted overnight and abstained from using tobacco, alcohol, caffeine, and vasoactive medications, we collected endothelial function parameters by using a digital thermal monitoring device approved by the US Food and Drug Administration (VENDYS-II; Endothelix, Inc., Houston, TX, USA). After letting the patients rest in a supine position for 30 min in a room with a temperature of 22 °C to 24 °C, blood pressure cuffs were put on their right upper arm, and skin temperature sensors were placed on bilateral index fingers (left, control; right, occlusion). Digital thermal monitoring of both hands was performed during 5-min stabilization, 5-min cuff inflation, and 5-min deflation. The right upper arm cuff was rapidly inflated to 50 mm Hg greater than systolic blood pressure for 5 min and then rapidly deflated to invoke a reactive hyperemic response in the fingertip distally. The more the temperature rebounded, the better the vascular reactivity. VRI has been defined as the maximal temperature difference between the rebound curve and the zero reactivity curves during the period of reactive hyperemia period, calculated by the VENDYS software. VRIs were found to range from 0.0 to 3.5; a VRI of less than 1.0 indicated poor vascular reactivity; a VRI ranging from 1.0 to 1.9, intermediate vascular reactivity; and a VRI of 2.0 or greater, good vascular reactivity [7,11].

### 2.5. Statistical Analysis

Data were calculated as means ± standard deviation (SD) and were assessed for normal distribution using the Kolmogorov-Smirnov test. Measured values between groups (poor, intermediate, and good VRI) were assessed using a Kruskal-Wallis analysis for parameters with non-normal distribution (fasting glucose, triglycerides, BUN, creatinine, and osteocalcin) and one-way analysis of variance for normally distributed data; then measured values were analyzed using Fisher’s protected t-test. Fasting glucose, triglycerides, BUN, creatinine, and osteocalcin levels were log-transformed to fulfill normality. Variables that correlated with VRI were examined using simple linear regression analyses, and significant variables were included in a multivariable forward stepwise regression analysis. A *p*-value of less than 0.05 was considered statistically significant. All statistical analyses were performed using the statistical package SPSS for Windows (Version 19.0; SPSS Inc., Chicago, IL, USA).

## 3. Results

The clinical characteristics and immunosuppressant drugs used by KT recipients are presented in Table 1. Of the 68 KT recipients, 8 (11.8%) had poor VRI, 26 (38.2%) had intermediate VRI, and 34 (50.0%) had good VRI. Significant between-group differences were noted in osteocalcin levels: levels in the groups with worse VRI were found to be higher (*p* = 0.001). Among the comorbid conditions, 31 patients (45.6%) were identified to have diabetes, and 26 (38.2%) had hypertension. Prescribed immunosuppressant drugs included tacrolimus, taken by 45 patients (66.2%); mycophenolate mofetil, taken by 43 patients (63.2%); steroids, taken by 59 patients (86.8%); rapamycin, taken by 4 patients (5.9%); and cyclosporine, taken by 14 patients (20.6%). Among the different groups, no significant differences were noted in the following factors as follows: gender; transplantation model; the presence of diabetes or hypertension; use of tacrolimus, mycophenolate mofetil, or mycophenolic acid; or the use of steroids, rapamycin, or cyclosporine.

Clinical characteristics and serum VRIs for the 68 KT recipients are presented in Table 2. Advanced age (*r* = −0.361, *p* = 0.002) and serum osteocalcin level (*r* = −0.432, *p* < 0.001) were found to be negatively correlated with VRI values in KT recipients. Forward stepwise linear regression analysis of the variables that were significantly associated with VRIs revealed that a low serum level of osteocalcin (β = −0.391, adjusted *R*^2^ change = 0.174, *p* < 0.001) and advanced age (β = −0.308, adjusted *R*^2^ change = 0.084, *p* = 0.005) were significantly and independently associated with VRIs in KT recipients.

## 4. Discussion

Our findings showed that increased serum osteocalcin level was associated with poor VRI, which was suggestive of endothelial dysfunction, and that this association was not dependent on the age of KT recipients.

Serum osteocalcin levels were observed to be elevated in patients with chronic kidney disease; this could be attributed to decreased renal clearance and increased bone metabolism. Increases in serum osteocalcin levels in parallel with kidney disease progression are closely related to iPTH levels [13,14]. After transplantation, osteocalcin level gradually decreases along with iPTH levels, and renal function is recovered [15]. Osteocalcin was considered a marker of bone turnover, inasmuch as it is released in its uncarboxylated form into the circulation after bone resorption by osteoclasts [13]. Moreover, osteocalcin acts like a hormone in modulating glucose metabolism; decreased levels of osteocalcin are often linked to diabetes mellitus and metabolic disease [16]. Some studies have demonstrated an inverse relationship between osteocalcin level and arterial calcification (carotid or coronary atherosclerosis) [16]. However, despite the seemingly favorable metabolic and cardiovascular effects associated with osteocalcin, it is also implicated in the pathogenesis of vascular calcification, which occurs in the chronic stage of atherosclerosis [17]. In bone formation, γ-carboxylated osteocalcin has been determined to bind to calcium and hydroxyapatite as part of bone mineralization [17]. In vascular calcification, hydroxyapatite is deposited in the arterial wall; this is driven by transdifferentiated vascular smooth muscle cells with an osteogenic phenotype [10]. In patients with coronary atherosclerosis, a higher percentage of endothelial progenitor cells have been identified to express osteocalcin [18]. Compelling evidence suggests that impaired bone metabolism is related to the development of vascular calcification; such evidence includes the finding of a higher prevalence of carotid atherosclerosis with higher osteocalcin levels and low bone mineral density in postmenopausal women [19]. Osteocalcin expression is induced in vascular smooth muscle cells, endothelial progenitor cells, and fibroblasts by endothelial cells activated by endothelial damage [20]. Endothelial dysfunction is an early feature of atherosclerotic vascular disease and significantly contributes to impairment of microvascular perfusion [21]. Therefore, osteocalcin is associated with atherosclerosis and endothelial dysfunction [17]. Our results demonstrated a significantly negative association between serum osteocalcin levels and VRIs in KT recipients, which suggests that osteocalcin plays a role in modulating endothelial dysfunction in KT recipients.

The real effect of osteocalcin on atherosclerosis and cardiovascular diseases remains unknown; the use of serum levels to predict cardiovascular events and mortality has yielded conflicting results. In one study, lower osteocalcin levels were associated with carotid atherosclerosis in patients with chronic kidney disease who were not receiving dialysis [22]. In another study, pulse wave velocity, a measure of arterial stiffness, was found to be associated inversely with serum osteocalcin levels in patients receiving chronic hemodialysis [9]. In a large-scale study involving 3542 community-dwelling older men, osteocalcin level was found to be predictive of all-cause and cardiovascular disease-related mortality in a U-shaped distribution, whereby increased rates of mortality were associated with either higher or lower levels of osteocalcin [23]. Similar findings of a U-shaped distribution of fatal events were reported in a large cohort of men at high risk for cardiovascular disease [24].

This study has some limitations. First, the study sample was relatively small, and the number of patients with poor vascular reactivity was the smallest of all the three groups. Second, this was a cross-sectional study, and thus we could not determine the causality. Third, we did not measure levels of uncarboxylated osteocalcin, which is shown by some studies to be the bioactive form of osteocalcin. Larger prospective studies are needed in the future to address these questions.

## 5. Conclusions

This study showed that higher serum osteocalcin level was associated with lower VRI and poorer endothelial dysfunction among KT recipients.

## Figures and Tables

**Table 1:** Clinical characteristics according to different vascular reactivity indices by digital thermal monitoring of 68 kidney transplantation patients.

Characteristics	All Patients (*n* = 68)	Good Vascular Reactivity (*n* = 34)	Intermediate Vascular Reactivity (*n* = 26)	Poor Vascular Reactivity (*n* = 8)	*p* Value
Age (years)	46.57 ± 10.51	45.44 ± 11.03	46.46 ± 10.15	51.75 ± 8.88	0.315
KT duration (months)	77.13 ± 52.60	71.79 ± 45.78	91.31 ± 56.82	53.75 ± 59.66	0.148
Height (cm)	161.27 ± 7.59	160.46 ± 6.90	160.92 ± 7.32	165.88 ± 10.36	0.185
Body weight (kg)	64.76 ± 13.13	65.92 ± 13.38	63.58 ± 12.78	63.66 ± 14.47	0.772
Body mass index (kg/m^2^)	24.85 ± 4.52	25.61 ± 5.12	24.44 ± 3.77	22.95 ± 3.65	0.276
Vascular reactivity index	1.91 ± 0.79	2.49 ± 0.53	1.60 ± 0.26	0.49 ± 0.31	< 0.001 *
Systolic blood pressure (mmHg)	144.81 ± 18.78	143.06 ± 18.31	146.15 ± 20.91	147.88 ± 14.14	0.731
Diastolic blood pressure (mmHg)	83.47 ± 12.52	83.82 ± 13.08	82.58 ± 12.76	84.88 ± 10.38	0.881
Total cholesterol (mg/dL)	191.81 ± 46.03	184.53 ± 36.81	201.69 ± 55.93	190.63 ± 45.80	0.363
Triglyceride (mg/dL)	121.00 (90.25–176.75)	117.00 (86.75–226.00)	127.00 (96.00–170.25)	119.00 (75.25–156.50)	0.878
Fasting glucose (mg/dL)	96.00 (88.00–113.75)	96.50 (87.00–109.25)	98.50 (88.00–141.75)	92.00 (87.50–99.00)	0.401
Blood urea nitrogen (mg/dL)	24.00 (16.00–34.75)	23.50 (16.00–28.25)	25.00 (15.50–42.75)	21.00 (15.00–38.25)	0.860
Creatinine (mg/dL)	1.40 (1.00–1.80)	1.30 (1.00–1.70)	1.55 (0.98–2.03)	1.35 (1.15–1.83)	0.458
eGFR (mL/min)	56.77 ± 24.45	59.25 ± 22.46	53.83 ± 28.16	55.75 ± 21.16	0.697
Total Calcium (mg/dL)	9.21 ± 0.65	9.18 ± 0.62	9.23 ± 0.71	9.27 ± 0.68	0.932
Phosphorus (mg/dL)	3.32 ± 0.83	3.28 ± 0.79	3.38 ± 0.91	3.24 ± 0.85	0.863
Osteocalcin (ng/mL)	6.73 (4.53–12.89)	5.96 (3.23–7.513)	11.03 (5.16–15.46)	12.13 (6.26–33.14)	< 0.001 *
Intact parathyroid hormone (pg/mL)	112.72 ± 82.16	98.16 ± 61.46	128.34 ± 105.92	123.86 ± 68.32	0.346
Female, *n* (%)	34 (50.0)	19 (55.9)	13 (50.0)	2 (25.0)	0.291
Diabetes mellitus, *n* (%)	31 (45.6)	16 (47.1)	14 (53.8)	1 (12.5)	0.118
Hypertension, *n* (%)	26 (38.2)	11 (32.4)	12 (46.2)	3 (37.5)	0.551
Living donor, *n* (%)	15 (22.1)	4 (11.8)	9 (34.6)	2 (25.0)	0.104
Tacrolimus use, *n* (%)	45 (66.2)	23 (67.6)	18 (69.2)	4 (50.0)	0.584
Mycophenolate mofetil use, *n* (%)	43 (63.2)	20 (58.8)	17 (65.4)	6 (75.0)	0.666
Steroid use, *n* (%)	59 (86.8)	30 (88.2)	23 (88.5)	6 (75.0)	0.579
Rapamycin use, *n* (%)	4 (5.9)	1 (2.9)	3 (11.5)	0 (0)	0.282
Cyclosporine use, *n* (%)	14 (20.6)	8 (23.5)	3 (11.5)	3 (37.5)	0.237

Values for continuous variables given as means ± standard deviation and test by one-way analysis of variance; variables not normally distributed given as medians and interquartile range and test by Kruskal-Wallis analysis; values are presented as number (%) and analysis after analysis by the chi-square test. eGFR, estimated glomerular filtration rate. * *p* < 0.05 was considered statistically significant after Kruskal-Wallis analysis or one-way analysis of variance.

**Table 2:** Correlation of vascular reactivity index levels and clinical variables by univariable or multivariable linear analyses among 68 kidney transplantation patients.

Variables	Vascular Reactivity Index				
	Simple Linear Regression		Multivariable Linear Regression		
	*r*	*p* Value	Beta	Adjusted R^2^ Change	*p* Value
Female	0.158	0.198	-	-	-
Diabetes mellitus	0.038	0.760	-	-	-
Hypertension	−0.140	0.256	-	-	-
Living donor	−0.182	0.137	-	-	-
Age (years)	−0.361	0.002 *	−0.308	0.084	0.005 *
KT duration (months)	−0.158	0.199	-	-	-
Height (cm)	−0.186	0.128	-	-	-
Body weight (kg)	0.003	0.979	-	-	-
Body mass index (kg/m^2^)	0.106	0.390	-	-	-
Systolic blood pressure (mmHg)	−0.235	0.055	-	-	-
Diastolic blood pressure (mmHg)	−0.049	0.689	-	-	-
Total cholesterol (mg/dL)	−0.137	0.264	-	-	-
Log-Triglyceride (mg/dL)	−0.027	0.828	-	-	-
Log-Glucose (mg/dL)	−0.069	0.575	-	-	-
Log-Blood urea nitrogen (mg/dL)	−0.132	0.284	-	-	-
Log-Creatinine (mg/dL)	−0.169	0.168	-	-	-
eGFR (mL/min)	0.180	0.142	-	-	-
Total Calcium (mg/dL)	−0.041	0.743	-	-	-
Phosphorus (mg/dL)	−0.015	0.905	-	-	-
Log-Osteocalcin (ng/mL)	−0.432	<0.001 *	−0.391	0.174	<0.001 *
iPTH (pg/mL)	−0.050	0.683	-	-	-

Data of triglyceride, fasting glucose, blood urea nitrogen, creatinine, and osteocalcin showed skewed distribution and therefore were log-transformed before analysis. Analysis of data was done using the simple linear regression analyses or multivariable stepwise linear regression analysis (adapted factors were age and log-osteocalcin). KT, kidney transplantation; eGFR, estimated glomerular filtration rate; iPTH, intact parathyroid hormone. * *p* < 0.05 was considered statistically significant.
